# Microwave Synthesized ZnO Nanorod Arrays for UV Sensors: A Seed Layer Annealing Temperature Study

**DOI:** 10.3390/ma9040299

**Published:** 2016-04-20

**Authors:** Ana Pimentel, Sofia Henriques Ferreira, Daniela Nunes, Tomas Calmeiro, Rodrigo Martins, Elvira Fortunato

**Affiliations:** i3N/CENIMAT, Department of Materials Science, Faculty of Science and Technology, Universidade NOVA de Lisboa, Campus de Caparica, Caparica 2829-516, Portugal; sofialhf@gmail.com (S.H.F.); danielasilvanunes@gmail.com (D.N.); tomasrcalmeiro@gmail.com (T.C.); rm@uninova.pt (R.M.)

**Keywords:** ZnO nanostructures, spin-coating, hydrothermal synthesis, microwave, UV sensors

## Abstract

The present work reports the influence of zinc oxide (ZnO) seed layer annealing temperature on structural, optical and electrical properties of ZnO nanorod arrays, synthesized by hydrothermal method assisted by microwave radiation, to be used as UV sensors. The ZnO seed layer was produced using the spin-coating method and several annealing temperatures, ranging from 100 to 500 °C, have been tested. X-ray diffraction (XRD), scanning electron microscopy (SEM), atomic force microscopy (AFM) and spectrophotometry measurements have been used to investigate the structure, morphology, and optical properties variations of the produced ZnO nanorod arrays regarding the seed layer annealing temperatures employed. After the growth of ZnO nanorod arrays, the whole structure was tested as UV sensors, showing an increase in the sensitivity with the increase of seed layer annealing temperature. The UV sensor response of ZnO nanorod arrays produced with the seed layer annealed temperature of 500 °C was 50 times superior to the ones produced with a seed layer annealed at 100 °C.

## 1. Introduction

Zinc oxide (ZnO) is an n-type semiconductor that has been extensively studied due to its unique properties, namely its direct and wide band gap (3.37 eV) and high exciton binding energy (60 meV) at room temperature [1]. These exceptional properties allow ZnO to behave as an efficient semiconductor and, therefore, to be applied in several functional devices, such as thin film transistors [2,3], light emitting diodes (LEDs) [4], UV/ozone sensors [5,6,7], biosensors [8,9], piezoelectric devices [10,11], among others.

ZnO can be easily synthesized into different morphologies, such as nanorods, nanotubes, nanoflowers, nanotetrapods, nanoribbons or nanoneedles, by adjusting the synthesis conditions and methods [4,12,13,14,15,16]. In particular, ZnO nanorods (NRs) have attracted much attention mainly due to their high surface-to-volume ratio and high sensitivity under ambient conditions, thus making them highly appealing for sensing applications [17,18,19].

ZnO NRs have been mostly synthesized by bottom up techniques, including chemical vapor deposition (CVD) [18], electrodeposition [20], pulsed laser deposition (PLD) [21], spray pyrolysis [22], and hydrothermal/solvothermal synthesis, which includes either conventional heating [23] or microwave radiation [24,25,26]. Recently, hydrothermal/solvothermal syntheses have been preferred to the other techniques, since they have proven to be low temperature and low cost methods, despite being versatile for producing ZnO nanoparticles. Moreover, these latter synthesis routes can be allied to facile and low cost techniques for the deposition of the seed layer, namely the spin-coating method [27], obtaining them in continuous and uniform arrays.

Microwave assisted synthesis represents an attractive alternative when compared to conventional heating, as it offers the opportunity to complete reactions in minutes. This celerity is possible due to the fact that microwaves transfer energy directly to the reactive species [28]. Moreover, this synthesis route has been used for other materials, forming homogeneous and uniform nanostructured films [29,30].

The present work reports the synthesis of ZnO nanorod arrays by the hydrothermal method assisted by microwave radiation, where the ZnO seed layer was deposited by spin-coating and glass was used as substrates. The microwave assisted synthesis conditions were kept constant. However, the effect of the annealing temperatures of the ZnO seed layer was investigated. The annealing temperatures tested ranged from 100 °C to 500 °C, and their effect on the seed layer was correlated to the final structural, morphological and optical properties of ZnO nanorods arrays. After an extensive characterization, the resulting ZnO nanorod arrays were tested as UV sensors.

## 2. Results and Discussion

### 2.1. Thermal Analysis

**Figure 1:**
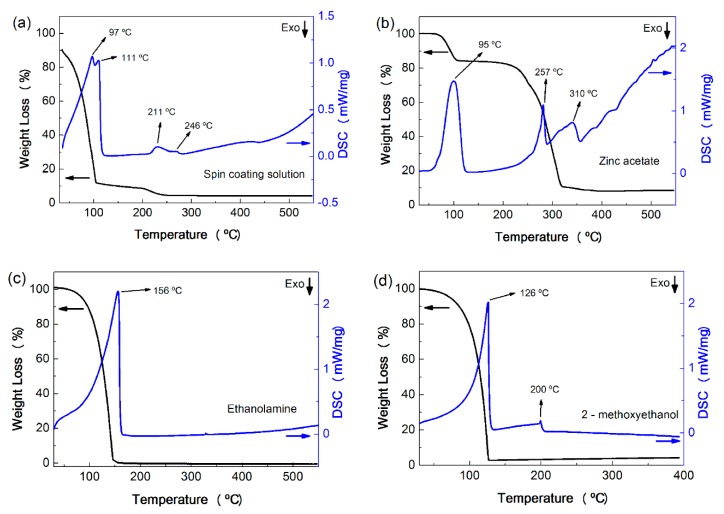
TG/DSC curves of the: (**a**) solution used in the formation of ZnO seed layer; (**b**) zinc acetate; (**c**) ethanolamine; and (**d**) 2-methoxyethanol.

In order to understand the influence on the annealing temperature on the final properties of the ZnO nanorod arrays, differential scanning calorimetric (DSC) measurements of their prior solutions, reactants and solvents, individually, were carried out (zinc acetate, ethanolamine and 2-methoxyethanol). The results are presented on Figure 1.

Ethanolamine presents a boiling point of 170 °C and a boiling interval of 69–70 °C (Figure 1c) and acts as a complexing agent, which retards the Zn^2+^ condensation; however, its presence also increases the pH, which promotes the formation of ZnO [31]. The use of 2-methoxyethanol, also with a high boiling point, around 125 °C (Figure 1d), will help the formation of aligned grain growth. Ohyama *et al.* [32] reported that solvents with low boiling points disturb the aligned grain growth in the film. Solvents with a high boiling point result in strongly preferential orientation of ZnO crystals, since they evaporate more slowly when heating, allowing the structural relaxation of the film before crystallization [33].

The DSC results of the solution used for the ZnO seed layer deposition, as well as their reactants and solvents are presented in Figure 1a. The DSC curve displayed a strong endothermic peak composed by two small peaks at 97 °C and 111 °C, which corresponds to the evaporation of the solvent in the solution (water and 2-methoxyethanol). This peak is accompanied by an expressive weight loss due to material evaporation. Between 200 °C and 250 °C, there are two small endothermic peaks, accompanied by a small weight loss (about 5%). These peaks can be correlated to the decomposition of 2-methoxyethanol, residual organics and the ethanolamine, that can occur between 204 °C and 232 °C [32]. Zinc acetate starts to dehydrate becoming anhydrous zinc acetate around 100 °C. Further decomposition of anhydrous zinc acetate from 150 to 280 °C (see Figure 1b) causes the formation of Zn_4_O(CH_3_CO_2_)_6_, which finally decomposes into ZnO [34]. By the thermal analysis of solution (Figure 1a), it is possible to observe that the final weight loss occurs between 200 °C and 250 °C attesting that no further decomposition occurred after this temperature. Since the boiling points of the solvents 2-methoxiethanol and ethanolamine are 125 °C and 170 °C, respectively, it can be concluded that annealing temperatures above 200 °C may be required for those to vaporize completely.

### 2.2. Structure and Morphology

**Figure 2:**
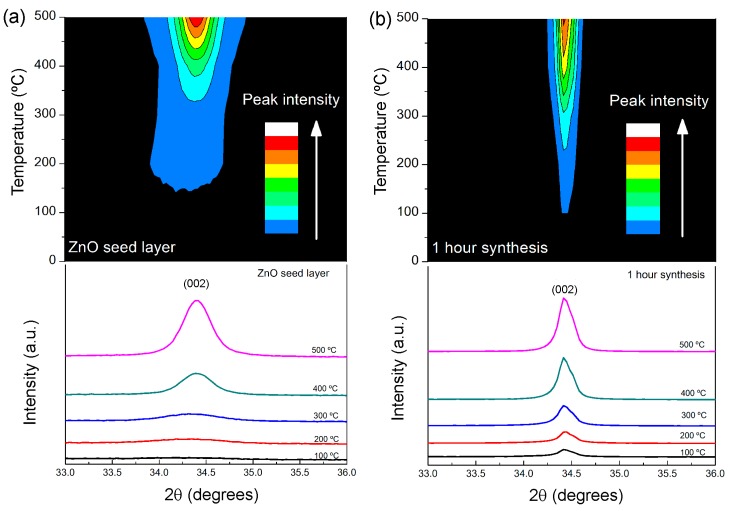
XRD diffractograms of: (**a**) ZnO thin films deposited by spin-coating; and (**b**) ZnO nanorod arrays grown by hydrothermal method assited by microwave radiation for 1 h.

In order to structurally characterize the ZnO nanorod arrays, XRD, SEM and AFM experiments were carried out. The ZnO nanorod synthesis was carried out with a microwave power input of 100 W and a constant temperature of 100 °C, for 60 min. Figure 2 presents XRD diffractograms of the ZnO materials (seed layers and nanorod arrays). A single peak can be observed at 2*θ* = 34.4°, which is fully assigned to plane (002) of hexagonal wurtzite ZnO structure and displaying lattice constants of a = 0.3296 nm and c = 0.5207 nm in accordance to Reference [14]. This result is attributed to the low surface energy of the (002) plane, which induced the growth of grains along this plane rather than along other orientation [35]. The results confirm that pure and very well aligned ZnO nanostructures were obtained.

The peak intensity of the (002) plane is found to increase with increasing annealing temperature, for seed layer and nanorod arrays, indicating that the degree of crystal orientation, as well as grain sizes increased with the increase of annealing temperature. The low intensity of (002) diffraction peak of seed layer annealed at 100 °C indicates that the thin film is nearly amorphous. This latter result is in consonance with Ohyama [32], which reported that the use of 2-methoxiethanol and ethanolamine, with high boiling points, will produce strongly preferred orientated ZnO nanocrystals. Higher annealing temperatures ensured higher preferred orientation regarding the (002) plane, which can be associated to the structural relaxation of the gel film before crystallization. However, the growth of ZnO nanorod arrays on amorphous thin film (annealed at 100 °C) resulted in a highly crystalline ZnO nanorods, meaning that the amorphous ZnO seed layer does not limit the growth along the (002) plane [27].

The grain sizes of ZnO thin films and nanorod arrays were calculated from Scherre’s equation [36]:

(1) D=0.94 λ / β cosθ

where *λ* is the wavelenght of the X-ray radiation; *θ* is the Bragg’s angle; and *β* is the full width at half maximum. In Table 1 are descriminated the grain sizes of the ZnO seed layers and ZnO nanorod arrays, for the different annealing temperatures. An increase in the annealing temperature generally leads to increase of crystallite size, which can be attributed to the increase of atoms mobility and consequently the increase of surface diffusion. These phenomena enable these atoms to migrate longer distances and occupy lattice sites with favorable stable energies. In turn, the crystallite size increase due to a surface energy minimization [37].

**Figure 3:**
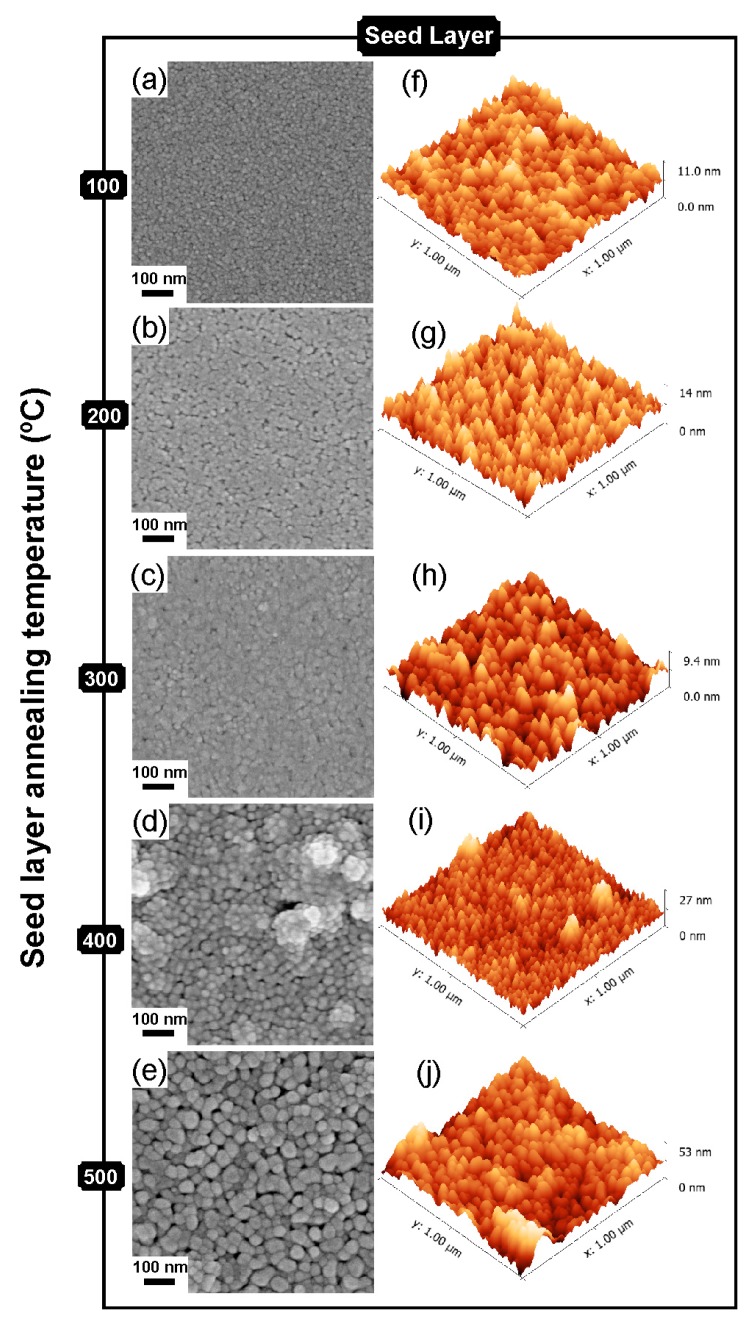
(**a**–**e**) SEM images; and (**f**–**j**) AFM images of ZnO seed layers produced with different annealing temperature (100 °C, 200 °C, 300 °C, 400 °C and 500 °C, respectively).

SEM and AFM measurements were carried out for the ZnO seed layers produced with different annealing temperatures and the surface topography images are presented in Figure 3. The ZnO seed layers showed an increase of roughness with the annealing temperature, where the appearance of nanostructures became discernible, for higher annealing temperatures. From 3D AFM images in Figure 3f–j, the roughness increase with temperature can be clearly seen. The roughness values were found to be 1.34 nm, 1.78 nm, 2.51 nm, 2.82 nm and 6.89 nm, for seed layers annealed at 100 °C, 200 °C, 300 °C, 400 °C and 500 °C, respectively. The increase of roughness can be associated with the growth of the crystals, thus being consistent with the XRD results.

**Figure 4:**
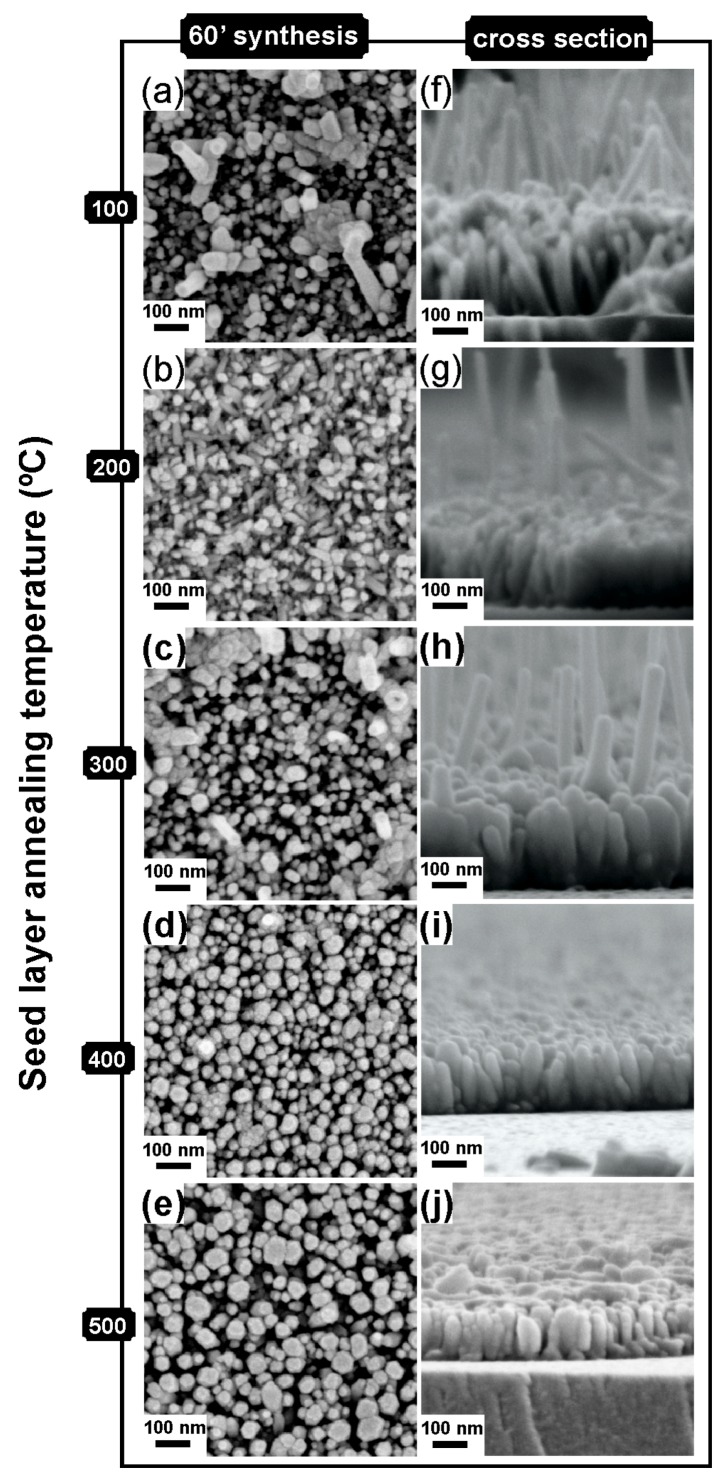
(**a**–**e**) SEM images; and (**f**–**j**) cross section images of ZnO nanorod arrays produced by hydrothermal method and assisted by microwave radiation, with different seed layer annealing temperature (100 °C, 200 °C, 300 °C, 400 °C and 500 °C, respectively).

Regarding the ZnO nanorod arrays synthesized by hydrothermal method and assisted by microwave radiation, the density and diameter of the nanorods clearly increase with the increase of annealing temperature (see Figure 4). It is also possible to observe that with the increase of annealing temperature, the ZnO nanorod arrays showed a more homogeneous growth when compared to lower temperatures used. This inhomogeneous growth of ZnO nanorods for lower annealing temperatures (up to 300 °C) can be confirmed with the SEM cross-section images (see Figure 4f–j), where it is possible to observe the presence of abnormal nanorods. It is also possible to observe that the columnar ZnO nanocrystals are well aligned along the substrate with the *c*-axis oriented perpendicular to the substrate surface. The ZnO nanorod length increased from about 180 to 350 nm with the increase of temperature from 100 to 300 °C. For higher temperature, the nanorod length decreased, however they became clearly thicker.

The increase in ZnO nanorod length is due to the increase of reactivity of the seed layer surface associated to the increase of annealing temperature. The ZnO crystal structure consist of alternating planes composed of coordinated Zn and O atoms along the *c*-axis direction. Usually the Zn-terminated polar (0001) plane is at the top surface of the ZnO crystal structure, whereas the O-terminated polar (0001) plane is at the bottom surface, causing a normal dipole moment and a polar surface of ZnO seed layer [24,38]. The presence of two charges (Zn^2+^ and OH^−^ ions) in the hydrothermal solution results in attraction toward the polar surface of ZnO seed layer. The reactivity of ZnO seed layer can be improved with the increase of annealing temperature, which will be stronger than the electrostatic interaction of the polar surface of the ZnO seed layer with the charges of Zn^2+^ and OH^−^. By this reason, more Zn^2+^ and OH^−^ ions are diffused on the ZnO seeds, which results in an increased ZnO nanorod length with increased ZnO seed layer annealing temperature [38]. However, further increase annealing temperature of the seed layer may result in shorter ZnO nanorods length that can be due to structural deterioration.

The increase of annealing temperature of ZnO seed layer will enhance the interaction of the grains, leading the grains to merge together to form rough ZnO seeds, resulting in larger diameters of the synthesized ZnO nanorods [39]. Thus, the grain size of ZnO seed layer is a key factor that will influence the nucleation of ZnO nanorod arrays.

### 2.3. Optical Properties

Optical band gaps of ZnO nanorod arrays ware evaluated from transmittance data through the well-known Tauc relation, for direct band semiconductors (Equation (2)) [40]:

(*αhν*)^m^ = A(*hν* − *E_g_*) (2)

where *α* is the absorption coefficient; A is an energy-independent constant; m is a constant that determines the type of the optical transition (m = 2 for allowed direct transitions and m = 1/2 for allowed indirect transitions), *E_g_* is the optical band gap, *h* is the Planck constant, and *ν* is the frequency.

**Figure 5:**
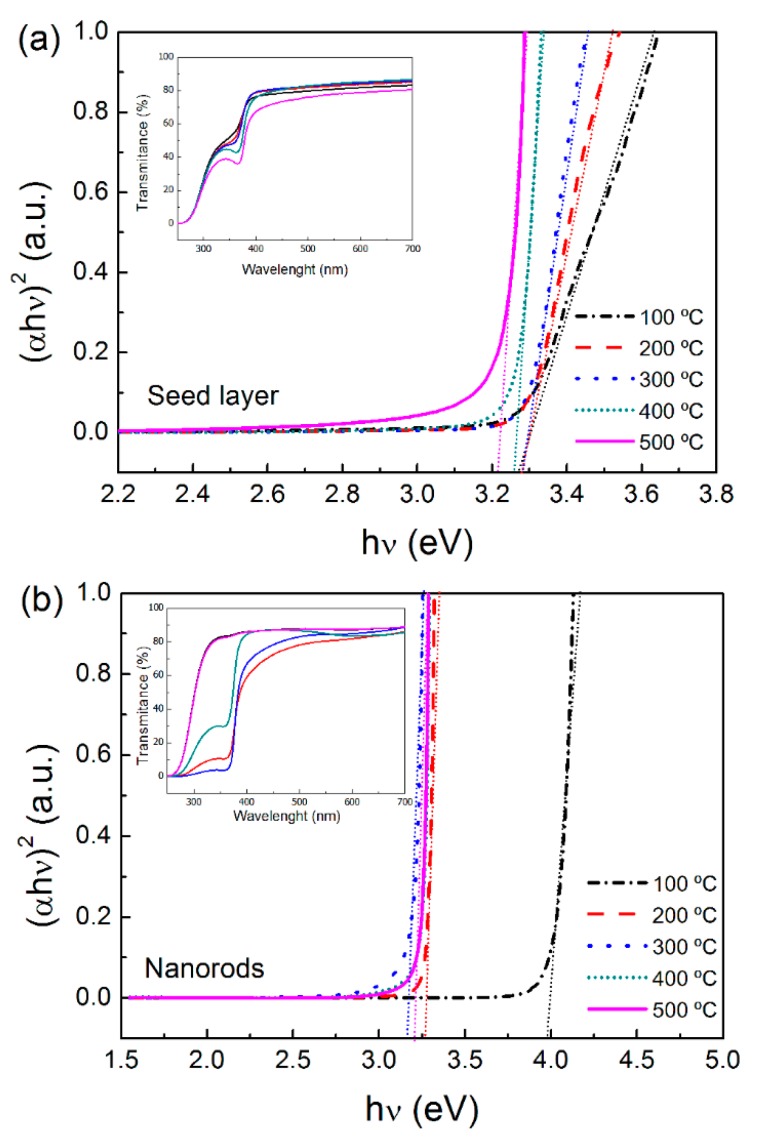
Band gap of: (**a**) ZnO seed layers at different annealing temperatures; and (**b**) ZnO nanorod arrays grown above these seed layers. The insets show the optical transmittance of the produced ZnO materials.

The optical band gap was obtained by extrapolating (*αhν*)^2^ *vs.* *hν* and the results are presented in Figure 5. The insets show the optical transmittance of the ZnO seed layers and the ZnO nanorod arrays. The ZnO nanostructured films were found to be transparent in the visible range, with an average transmittance between 75% and 80%. Such high transmittance of the ZnO seed layers is a consequence of his low thickness (~50 nm) and small and highly connected ZnO grains. After the growth of ZnO nanorods, where the thickness increases to 180–300 nm, the transmittance remained the same. The high transparency of ZnO nanorod array is due to their high columnar oriented grains and for being well-aligned perpendicular to the substrate. These results corroborate the SEM surface and cross-section images.

The estimated band gaps of ZnO seed layers were found to be 3.30 eV, for annealing temperatures of 100, 200, and 300 °C, and 3.26 and 3.22 eV for annealing temperatures of 400 and 500 °C, respectively. The ZnO seed layer band gap decreased for higher annealing temperatures, which is in accordance with previous reports that showed that this effect of the annealing temperature on the optical band gap is associated with the increase of crystallites size [41].

In the case of ZnO nanorod arrays, band gap values were 3.98, 3.27, 3.17, 3.20 and 3.20 eV for 100, 200, 300, 400, and 500 °C, respectively. It is observed that the band gap initially decreased (for temperatures from 100 to 300 °C) followed by a slightly increase for higher temperatures (for 400 and 500 °C).

There are several parameters that may influence the optical band gap of a semiconductor material, like the residual strain, defects and grain size confinement. It is well known that the band gap usually decreases with the increase of grain size and thickness, which were not observed in these ZnO nanorod arrays. The increase in the energy band gap with the increase annealing temperature observed for higher temperatures (400 and 500 °C), may be attributed to an expected diminution of oxygen vacancy defects in the ZnO nanostructures, hence diminishing the density of mid-gap states induced by oxygen vacancies [42].

### 2.4. ZnO Nanorods Array UV Sensor

Ultraviolet (UV) is a high energy beam with photon energies varying from 3.0 to 12.4 eV (400–10 nm), which overlaps with the band gap of zinc oxide, around 3.4 eV [43]. The use of nanostructures for sensing applications have the advantage of a high surface area and electronic processes are strongly influenced by surface processes [13]. The sensing process is governed by the oxygen vacancies on the surface that influences the electronic properties of ZnO. Upon oxidation, with the adsorption of molecules containing a high concentration of O_2_ at vacancy sites that accept electrons, electrons are withdrawn and effectively depleted from the conduction band leading to reduction of conductivity. On the other hand, reducing molecules can react with surface adsorbed oxygen leaving behind an electron and a higher conductivity [13]. In consequence, the exposure to UV radiation is an efficient way to excite the hole-electron pairs in the sensing materials, such that the measured resistance of the semiconductor metal oxides is significantly reduced [43].

In the dark, the oxygen molecules adsorb on the ZnO nanorod surface and capture the free electrons present in the n-type oxide semiconductor (Equation (3)) and a low conductive depletion layer is formed near the surface, which results in the reduction of the conduction layer. When the ZnO nanorods are irradiated with UV radiation, with photon energy above ZnO band-gap energy (*Eg*), the electron-hole pairs are photogenerated (Equation (4)) and the holes migrate to the surface along the potential slope. This potential slope is produced by band bending and the discharge of the negatively charged adsorbed oxygen ions through surface electro-hole recombination (Equation (5)). Consequently, oxygen is photodesorbed from surface [44].

(3) $$O_{2}\left(g\right)+\;e^{-}\;\to \;O_{2}^{-}\left(ad\right)$$

(4) $$h\nu \;\to \;e^{-}+\;h^{+}$$

(5) $$h^{+}+\;O_{2}^{-}\left(ad\right)\;\to \;O_{2}\left(g\right)$$

**Figure 6:**
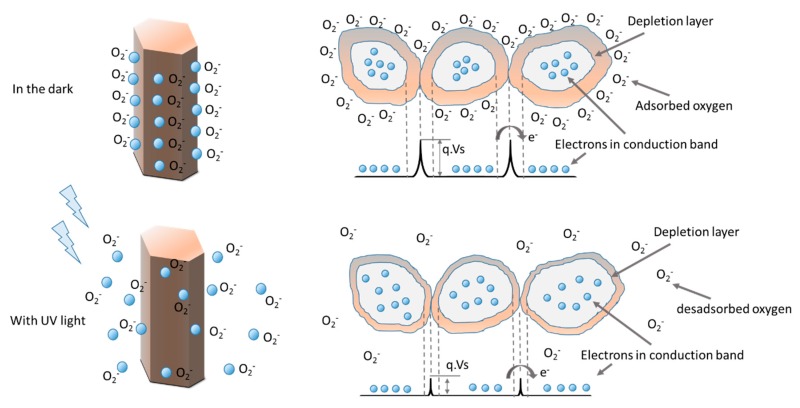
Schematic of the relation between the band model and barrier at intergranular ZnO nanoparticles with the UV off and the UV on [45].

A schematic of these reactions can be observed in Figure 6.

The sensitivity of nanostructured ZnO as a sensor element is comparatively high due to the grain-size effect [46]. It is well known that the sensing mechanism of ZnO belongs to the surface-controlled type. The sensitivity variation is related to the grain size, geometry, surface state, oxygen adsorption quantity, active energy of oxygen adsorption and lattice defects [46]. Usually, the smaller the grain size, specific surface area and oxygen adsorption quantity, the higher is the sensitivity of a sensor. However, for nano-grain based sensors, the sensing properties are influenced not only by the microstructural features, such as the grain size, the geometry, but also by the connectivity between the grains and grains defects.

**Figure 7:**
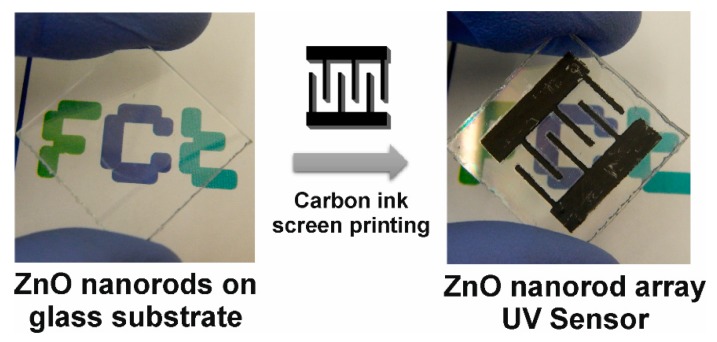
Photograph of the ZnO nanorod array UV sensor with contacts produced by screen printed of carbon ink.

To characterize the ZnO nanorod arrays as a UV sensor, interdigital contacts were screen printed on top of the ZnO seed layer with the grown nanorod arrays (see scheme in Figure 7). The ZnO nanorod arrays were then subjected to irradiation cycles of ultraviolet radiation.

**Figure 8:**
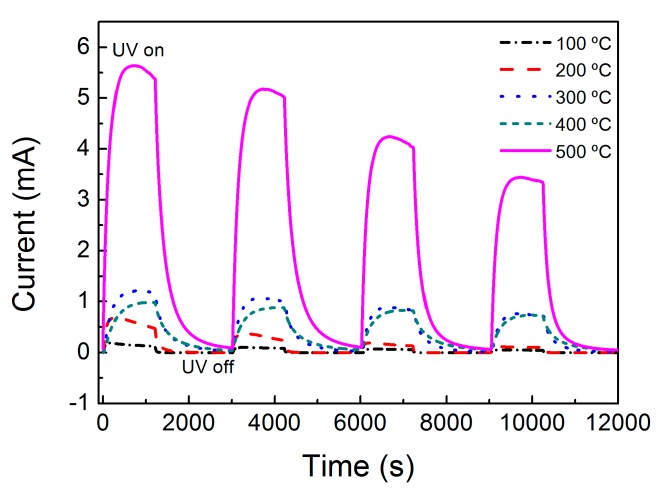
Cycling behaviour of ZnO nanorods based UV sensors.

Figure 8 shows the time resolved photocurrent of ZnO nanorods UV sensor, produced with different annealing temperatures, in response to turning on/off the UV radiation, at room temperature. It can be seen that the ZnO nanorods array sensor produced with an annealing temperature of 500 °C displayed enhanced sensing performance. Under the bias voltage of 5 V, the photocurrent exponential increased from 0.5 µA to 0.1 mA for the material with an annealing temperature of 100 °C and up to 5.5 mA for the one produced with an annealing temperature of 500 °C, within about 60 s, with saturation in the on-state. After the UV radiation was turned off, the current decreased again to the initial value of current.

The values of dark currents and photocurrents of the produced materials were measured and are presented in Table 2.

The responsivity, *R*, of the fabricated UV sensors can be calculated according to Equation (6) [47]:

(6) $$R=\frac{I_{ph}-I_{dark}}{P_{UV}}$$

where *I_ph_* is the UV sensor photocurrent; *I_dark_* is the UV sensor dark current; and *P_UV_* is the power of the UV source. For the ZnO nanorod arrays UV sensor, produced with different seed layer annealing temperatures, the calculated responsivity was 0.008 mA/W, 0.03 mA/W, 0.07 mA/W, 0.08 mA/W and 0.4 mA/W, for annealing temperatures of 100 °C, 200 °C, 300 °C, 400 °C and 500 °C, respectively.

Thus, the ZnO nanorod array UV sensor, produced with a seed layer subjected to an annealing temperature of 500 °C, presents a photo response 50 times superior when compared with sample produced with a seed layer annealing temperature of 100 °C.

The higher sensitivity for the material annealed at 500 °C can be related to the reduction of the surface-defect-related trap centers that results from the increase of the seed layer annealing temperature, hence modifying the quality of the ZnO nanorods grown by hydrothermal method and assisted by microwave radiation, resulting in a improvement of their UV response [48]. The ZnO nanorods produced using seed layers with a higher annealing temperature will present higher cystalline content (proved by the XRD results) and less structural defects.

The produced ZnO nanorod array UV sensors presents low values of decay time after the UV radiation is turned off, and this decay is related with the evacuation of the charge carries. It is expected that the films annealed at higher temperatures presents a lower decay value due to the annihilation of surface trap [49].

Several factors may be responsible for the high values obtained for decay time, such as the annealing temperature, the thickness of nanorods and even the type and design of electrical contacts that are used. For this sort of sensors, as the main goal is to use low cost techniques, the contacts used were based of carbon ink and deposited by screen printing technique. In such approach, only part of the nanorods (the top) is kept in contact with the ink (that is not so conductive as an aluminum or silver contact). This can lead to the photogenerated carriers to be kept trapped within the ZnO nanorods for longer period of time before being injected into the carbon contacts, and thus leading to a slow saturation in photocurrent and slow decay when the UV radiation is turned off [50]. This may be the probable cause of having sensors with a long decay response. The layout of the contacts can be improved in order to reduce the UV sensor decay time.

## 3. Experimental Details

### 3.1. Synthesis of ZnO Nanostructures

The ZnO thin films to serve as a seed were deposited on glass substrate by spin-coating method. A coating solution was prepared from zinc acetate dihydrate (Zn(CH_3_COO)_2_·2H_2_O; 98%, CAS: 5970-45-6), ethanolamine, MEA (C_2_H_7_NO; 99%, CAS: 141-43-5) and 2-methoxiethanol (C_3_H_8_O_2_, 99.8%, CAS: 109-86-4), all from Sigma-Aldrich (St. Louis, MO, USA). The reagents were used without further purification. In a typical experiment, zinc acetate solution was prepared by dissolving Zn(CH_3_COO)_2_·2H_2_O in 2-methoxiethanol and adding the ethanolamine. The concentration of the solution was chosen to be 0.35 M, in a proportion of 1:1 of zinc acetate and ethanolamine. The resulting solution was then stirred for 1 h at 60 °C and filtered afterward to yield a clear and homogeneous solution and then used for preparing films by spin-coating method. Before deposition, 20 × 20 mm^2^ glass substrates were successively cleaned with acetone, ethanol, and deionized water in an ultrasonic bath. The prepared solution was then spin coated on the substrate at 3000 rpm for 35 s at room temperature. After deposition, the films were dried for 10 min in a hot plate at 300 °C in order to remove the used solvent. After repeating the coating-drying cycles 4 times the substrates were annealed for 1 h. The annealing temperature was varied from 100 to 500 °C in order to investigate its effect in the resulting ZnO nanorod arrays.

**Figure 9:**
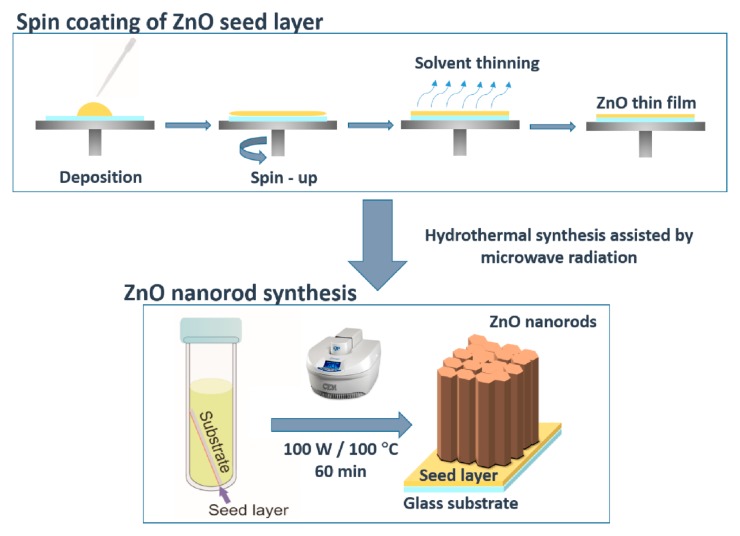
Scheme of the ZnO seed layer deposition using the spin coating method, followed by ZnO nanorod arrays synthesized by hydrothermal method assisted by microwave radiation.

After uniformly coating the glass substrates with ZnO thin films, ZnO nanorod arrays were synthesized by hydrothermal method assisted by microwave radiation. The ZnO seeded substrates (20 × 20 mm^2^) were placed at an angle against the vessel, with the seed layer facing down [51] (see Figure 9) and filled with an aqueous solution of 25 mM zinc nitrate hexahydrate (Zn(NO_3_)_2_·6H_2_O; 98%, CAS: 10196-18-6) and 25 mM hexamethylenetetramine (C_6_H_12_N_4_)_2_; 99%, CAS: 100-97-0), both from Sigma Aldrich (St. Louis, MO, USA).

The ZnO nanorod synthesis was carried out with a microwave power input of 100 W and a constant temperature of 100 °C, for 60 min. After each synthesis process, the materials were cleaned with deionized water and dried with compressed air. Figure 9 represents a schematic of the production process of ZnO seed layer and the subsequent synthesis of ZnO nanorod arrays.

### 3.2. Characterization Techniques

Differential scanning calorimetric measurements (DSC) of spin coating solution were carried out with a Simultaneous Thermal Analyser (TGA-DSC-STA 449 F3 Jupiter, Netzsch-Geratebau GmbH, Selb, Germany). Approximately 20 mg of each sample was loaded into an open aluminum crucible and heated from room temperature to 550 °C with a heating rate of 5 °C·min^−1^, in air.

The crystallinity of the as-prepared materials was determined using a PANalytical’s X’Pert PRO MRD X-ray diffractometer (PANalytical B.V., Almelo, The Netherlands), with a monochromatic CuK*α* radiation source (wavelength 1.540598 Å). XRD measurements were carried out from 30° to 40° (2*θ*), with a scanning step size of 0.016°. The morphology of ZnO seed layer and nanorods were characterized by SEM-FIB using a Carl Zeiss AURIGA CrossBeam workstation instrument (Carl Zeiss Microscopy GmbH, Oberkochen, Germany) equipped with an Oxford X-ray Energy Dispersive Spectrometer (Carl Zeiss Microscopy GmbH, Oberkochen, Germany).

Atomic force microscopy (AFM) was used to measure the roughness of the seed layers, using an MFP-3D stand alone Asylum Research instrument (Asylum Research Oxford Instruments, Goleta, CA, USA) operated in tapping mode using Olympus AC160TS silicon probes (Olympus Corporation, Tokyo, Japan) attached to cantilevers with a nominal quality factor of 550, spring constant of 26.1 N/m and frequency resonance peak at 300 kHz. The scanning range was 1 × 1 µm^2^ with a resolution of 256 × 256 lines. 

The transmittance spectra was obtained in the 250–800 nm range using a Perkin Elmer lambda 950 UV/VIS/NIR spectrophotometer (Perkin Elmer, Inc., Waltham, MA, USA). The band gap of ZnO was estimated from obtained transmittance spectra using the Tauc plot method [52,53].

### 3.3. Characterization of ZnO Nanorod Arrays as a UV Sensor

The synthesized ZnO nanorod arrays were characterized as a UV sensor, using a potentiostat model 600, from Gamry Instruments, Inc. (Warminster, PA, USA), in a chronoamperiometry configuration, with a constant applied voltage of 5 V. For interdigital electrical contacts, a carbon resistive ink, PE-C-774, from Conductive Compounds (Hudson, NH, USA), was used. The ZnO nanorod arrays were subjected to UV irradiation with an ultraviolet lamp, model TK-2028 (Hongguang Optics International Industry Co. Ltd., Ningbo, China) with an intensity of 6 W at a wavelength of 254 nm. The produced sensor was irradiated for 20 min followed by 20 min in off state.

## 4. Conclusions

The present work investigated the effect of the annealing temperature of the seed layer on the growth of ZnO nanorod arrays produced under hydrothermal synthesis assisted by microwave radiation and its direct influence on the final UV sensor performance. It has been demonstrated the successfully growth of ZnO nanorods, forming oriented nanorod arrays aligned along the (002) plane. The ZnO seed layer and nanorod arrays were observed by AFM and SEM revealing growth homogeneity and roughness with the increase of seed layer annealing temperature. All the ZnO nanorod arrays were tested as UV sensors, showing an increase in the sensitivity with the increase of seed layer annealing temperature. The UV sensor response of ZnO nanorods produced with a seed layer annealed at 500 °C was 50 times superior to those produced with a seed layer annealed at 100 °C. This work demonstrated that the ZnO seed layer annealing temperature, produced with a low cost method, have strong influence on the ZnO nanorods growth and on their sensing properties, by affecting the ZnO grains microstructure and their crystalographic defects. Moreover, it can be concluded that by using microwave assisted synthesis to grow ZnO nanorod arrays on seeded films annealed in a wide range of temperatures, it is possible to grow ZnO nanorods arrays and produce UV sensors in all type of substrates, from silicon and glass (that support high temperatures) to polymeric or paper substrates (that only support low temperatures).

## Figures and Tables

**Table 1:** Crystallite sizes of ZnO thin films (seed layers) and ZnO nanorod arrays, obtained with Scherrer’s equation.

Annealing Temperature	100 °C	200 °C	300 °C	400 °C	500 °C
Seed Layer (nm)	9.426	11.055	13.388	23.449	25.725
MW 60 min (nm)	38.621	40.779	47.235	54.972	56.274

**Table 2:** Dark currents and photocurrents of the produced materials.

Annealing Temperature	100 °C	200 °C	300 °C	400 °C	500 °C
Dark current (A)	5.0 × 10^−7^	1.0 × 10^−6^	3.0 × 10^−5^	4.0 × 10^−5^	9.0 × 10^−5^
Photocurrent (A)	1.0 × 10^−4^	3.7 × 10^−4^	8.7 × 10^−4^	1.0 × 10^−3^	5.0 × 10^−3^

## References

[1] Coleman V.A., Jagadish C., Jagadish C., Pearton S. (2006). Basic Properties and Applications of ZnO. Zinc Oxide Bulk, Thin Films and Nanostructures.

[2] Barquinha P., Fortunato E., Gonçalves A., Pimentel A., Marques A., Pereira L., Martins R. (2006). A Study on the Electrical Properties of ZnO Based Transparent TFTs. Mater. Sci. Forum.

[3] Fortunato E., Gonçalves A., Marques A., Pimentel A., Barquinha P., Águas H., Pereira L., Raniero L., Gonçalves G., Ferreira I. (2006). Multifunctional Thin Film Zinc Oxide Semiconductors: Application to Electronic Devices. Mater. Sci. Forum.

[4] Djurišić A.B., Ng A.M.C., Chen X.Y. (2010). ZnO nanostructures for optoelectronics: Material properties and device applications. Prog. Quantum Electron..

[5] Pimentel A., Gonçalves A., Marques A., Martins R., Fortunato E. (2006). Role of the thickness on the electrical and optical performances of undoped polycrystalline zinc oxide films used as UV detectors. J. Non Cryst. Solids.

[6] Pimentel A.C., Gonçalves A., Marques A., Martins R., Fortunato E. (2006). Zinc Oxide Thin Films used as an Ozone Sensor at Room Temperature. MRS Proc..

[7] Bai S., Wu W., Qin Y., Cui N., Bayerl D.J., Wang X. (2011). High-Performance Integrated ZnO Nanowire UV Sensors on Rigid and Flexible Substrates. Adv. Funct. Mater..

[8] Arya S.K., Saha S., Ramirez-Vick J.E., Gupta V., Bhansali S., Singh S.P. (2012). Recent advances in ZnO nanostructures and thin films for biosensor applications: Review. Anal. Chim. Acta.

[9] Zhang Y., Kang Z., Yan X., Liao Q. (2015). ZnO nanostructures in enzyme biosensors. Sci. China Mater..

[10] Kumar B., Kim S.-W. (2012). Energy harvesting based on semiconducting piezoelectric ZnO nanostructures. Nano Energy.

[11] Wang Z.L., Song J. (2006). Piezoelectric nanogenerators based on zinc oxide nanowire arrays. Science.

[12] Wang Z.L. (2004). Zinc oxide nanostructures: Growth, properties and applications. J. Phys. Condens. Matter.

[13] Schmidt-Mende L., MacManus-Driscoll J.L. (2007). ZnO—Nanostructures, defects, and devices. Mater. Today.

[14] Baruah S., Dutta J. (2009). Hydrothermal growth of ZnO nanostructures. Sci. Technol. Adv. Mater..

[15] Bai S., Chen L., Li D., Yang W., Yang P., Liu Z., Chen A., Liu C.C. (2010). Different morphologies of ZnO nanorods and their sensing property. Sens. Actuators B Chem..

[16] Spencer M.J.S. (2012). Gas sensing applications of 1D-nanostructured zinc oxide: Insights from density functional theory calculations. Prog. Mater. Sci..

[17] Wang L., Kang Y., Liu X., Zhang S., Huang W., Wang S. (2012). ZnO nanorod gas sensor for ethanol detection. Sens. Actuators B Chem..

[18] Lupan O., Emelchenko G.A., Ursaki V.V., Chai G., Redkin A.N., Gruzintsev A.N., Tiginyanu I.M., Chow L., Ono L.K., Roldan Cuenya B. (2010). Synthesis and characterization of ZnO nanowires for nanosensor applications. Mater. Res. Bull..

[19] Pimentel A., Nunes D., Duarte P., Rodrigues J., Costa F.M., Monteiro T., Martins R., Fortunato E. (2014). Synthesis of Long ZnO Nanorods under Microwave Irradiation or Conventional Heating. J. Phys. Chem. C.

[20] Wu L., Song F., Fang X., Guo Z.-X., Liang S. (2010). A practical vacuum sensor based on a ZnO nanowire array. Nanotechnology.

[21] Ajimsha R.S., Manoj R., Aneesh P.M., Jayaraj M.K. (2010). Violet luminescence from ZnO nanorods grown by room temperature pulsed laser deposition. Curr. Appl. Phys..

[22] Shinde S.D., Patil G.E., Kajale D.D., Gaikwad V.B., Jain G.H. (2012). Synthesis of ZnO nanorods by spray pyrolysis for H_2_S gas sensor. J. Alloys Compd..

[23] Gurav K.V., Gang M.G., Shin S.W., Patil U.M., Deshmukh P.R., Agawane G.L., Suryawanshi M.P., Pawar S.M., Patil P.S., Lokhande C.D. (2014). Gas sensing properties of hydrothermally grown ZnO nanorods with different aspect ratios. Sens. Actuators B Chem..

[24] Pimentel A., Rodrigues J., Duarte P., Nunes D., Costa F.M., Monteiro T., Martins R., Fortunato E. (2015). Effect of solvents on ZnO nanostructures synthesized by solvothermal method assisted by microwave radiation: A photocatalytic study. J. Mater. Sci..

[25] Hassan J.J., Mahdi M.A., Chin C.W., Abu-Hassan H., Hassan Z. (2013). Room temperature hydrogen gas sensor based on ZnO nanorod arrays grown on a SiO_2_/Si substrate via a microwave-assisted chemical solution method. J. Alloys Compd..

[26] Rai P., Song H.-M., Kim Y.-S., Song M.-K., Oh P.-R., Yoon J.-M., Yu Y.-T. (2012). Microwave assisted hydrothermal synthesis of single crystalline ZnO nanorods for gas sensor application. Mater. Lett..

[27] Huang J.-S., Lin C.-F. (2008). Influences of ZnO sol-gel thin film characteristics on ZnO nanowire arrays prepared at low temperature using all solution-based processing. J. Appl. Phys..

[28] Hayes B.L. (2002). Microwave Synthesis: Chemistry at the Speed of Light.

[29] Nunes D., Santos L., Duarte P., Pimentel A., Pinto J.V., Barquinha P., Carvalho P.A., Fortunato E., Martins R. (2015). Room temperature synthesis of Cu_2_O nanospheres: Optical properties and thermal behavior. Microsc. Microanal..

[30] Gonçalves A., Resende J., Marques A.C., Pinto J.V., Nunes D., Marie A., Goncalves R., Pereira L., Martins R., Fortunato E. (2016). Smart optically active VO_2_ nanostructured layers applied in roof-type ceramic tiles for energy efficiency. Sol. Energy Mater. Sol. Cells.

[31] Ohyama M., Kouzuka H., Yoko T. (1997). Sol-gel preparation of ZnO films with extremely preferred orientation along (002) plane from zinc acetate solution. Thin Solid Films.

[32] Ohyama M., Kozuka H., Yoko T., Sakka S. (1996). Preparation of ZnO films with preferential orientation by sol-gel method. J. Ceram. Soc. Jpn..

[33] Znaidi L. (2010). Sol-gel-deposited ZnO thin films: A review. Mater. Sci. Eng. B.

[34] Lin C.-C., Li Y.-Y. (2009). Synthesis of ZnO nanowires by thermal decomposition of zinc acetate dihydrate. Mater. Chem. Phys..

[35] Khomyak V.V., Slyotov M.M., Shtepliuk I.I., Lashkarev G.V., Slyotov O.M., Marianchuk P.D., Kosolovskiy V.V. (2013). Annealing effect on the near-band edge emission of ZnO. J. Phys. Chem. Solids.

[36] Cullity B.D. (1956). Elements of X ray Diffraction.

[37] Hoon J.-W., Chan K.-Y., Krishnasamy J., Tou T.-Y., Knipp D. (2011). Direct current magnetron sputter-deposited ZnO thin films. Appl. Surf. Sci..

[38] Ridhuan N.S., Razak K.A., Lockman Z., Abdul Aziz A. (2012). Structural and morphology of ZnO nanorods synthesized using ZnO seeded growth hydrothermal method and its properties as UV sensing. PLoS ONE.

[39] Baruah S., Dutta J. (2009). Effect of seeded substrates on hydrothermally grown ZnO nanorods. J. Sol Gel Sci. Technol..

[40] Talebian N., Amininezhad S.M., Doudi M. (2013). Controllable synthesis of ZnO nanoparticles and their morphology-dependent antibacterial and optical properties. J. Photochem. Photobiol. B.

[41] Hong R., Huang J., He H., Fan Z., Shao J. (2005). Influence of different post-treatments on the structure and optical properties of zinc oxide thin films. Appl. Surf. Sci..

[42] Panda S.K., Jacob C. (2012). Preparation of transparent ZnO thin films and their application in UV sensor devices. Solid State Electron..

[43] Chou C.-S., Wu Y.-C., Lin C.-H. (2014). Oxygen sensor utilizing ultraviolet irradiation assisted ZnO nanorods under low operation temperature. RSC Adv..

[44] Zhai T., Fang X., Liao M., Xu X., Zeng H., Yoshio B., Golberg D. (2009). A Comprehensive Review of One-Dimensional Metal-Oxide Nanostructure Photodetectors. Sensors.

[45] Madou M.J., Morrison S.R. (1989). Chemical Sensing with Solid State Devices.

[46] Fryxell G.E., Cao G. (2007). Environmental Applications of Nanomaterials: Synthesis, Sorbents and Sensors.

[47] Mamat M.H., Khusaimi Z., Zahidi M., Mahmood M.R., Yaln O. (2012). Nanorods.

[48] Zhou Q., Chen W., Xu L., Peng S. (2013). Hydrothermal synthesis of various hierarchical ZnO nanostructures and their methane sensing properties. Sensors.

[49] Mamat M.H., Khalin M.I.C., Mohammad N.N.H.N., Khusaimi Z., Sin N.D.M., Shariffudin S.S., Zahidi M.M., Mahmood M.R. (2012). Effects of Annealing Environments on the Solution-Grown, Aligned Aluminium-Doped Zinc Oxide Nanorod-Array-Based Ultraviolet Photoconductive Sensor. J. Nanomater..

[50] Chang H., Sun Z., Ho K.Y.-F., Tao X., Yan F., Kwok W.-M., Zheng Z. (2011). A highly sensitive ultraviolet sensor based on a facile *in situ* solution-grown ZnO nanorod/graphene heterostructure. Nanoscale.

[51] Nunes D., Pimentel A., Pinto J.V., Calmeiro T.R., Nandy S., Barquinha P., Pereira L., Carvalho P.A., Fortunato E., Martins R. (2015). Photocatalytic behavior of TiO_2_ films synthesized by microwave irradiation. Catal. Today.

[52] Pankove J.I. (1971). Optical Processes in Semiconductors.

[53] Tauc J. (1968). Optical properties and electronic structure of amorphous Ge and Si. Mater. Res. Bull..

